# Colorectal cancer survivorship program at a single tertiary centre: has service provision changed after COVID-19?

**DOI:** 10.1007/s00520-025-09762-6

**Published:** 2025-07-22

**Authors:** Rachael Menadue, Lea Tiffany, Shriranshini Satheakeerthy, Aditya Sakalkale, Matthew Wei, Fiona Reid, Ian Faragher, Justin M. Yeung

**Affiliations:** 1 Department of Colorectal Surgery, Western Health, Melbourne, Australia; 2 Department of Surgery, Western Precinct, University of Melbourne, Melbourne, Australia

**Keywords:** COVID-19, Colorectal cancer, Surveillance

## Abstract

**Background:**

Surveillance after colorectal cancer (CRC) resection is an important aspect of survivorship care. This study aimed to assess whether there were any changes to post-operative surveillance uptake in non-metastatic CRC patients and pre- and post-COVID pandemic in Victoria.

**Methods:**

All CRC patients (Stages I–III) who underwent curative surgery at Western Health, Victoria, Australia, were included. Surveillance included a three-monthly clinical review and carcinoembryonic antigen (CEA) up to 18 months and CT imaging and colonoscopy at 12 months following surgical resection.

**Results:**

Between 2019 and 2022, 380 patients were identified. Stage III patients had the highest uptake with regard to clinical reviews, CEA testing and 12-month CT (83.3%, 60.3% and 85.5%, respectively) while Stage I patients had the lowest (52.7%, 35.7% and 75.5% respectively) (*p* < 0.05). Colonoscopy surveillance was low regardless of stage (66.3%, 59.8% and 59.7% of Stages I, II and III, respectively). Uptake of CEA, clinic reviews and colonoscopy did not vary during our study period. More patients underwent 12-month CT following the COVID pandemic (87%) compared to pre-COVID (73.1%) or during COVID (76%, *p* < 0.05). There was no difference in 18-month mortality and overall recurrence during our study timelines.

**Conclusion:**

Lower-stage CRC patients had lower rates of surveillance uptake, in particular, with CEA blood tests and colonoscopy. Survivorship provision did not change pre- or post-COVID.

## Introduction

Colorectal cancer (CRC) remains the most common digestive tract malignancy in Australia and the second leading cause of cancer death [1]. Surveillance following curative resection for CRC is an essential aspect of survivorship care, as patients remain at risk of developing recurrence, most commonly within the first 2 years of surgery [2]. The incidence of CRC is increasing, with over 17,000 new cases diagnosed annually in Australia, resulting in more patients entering follow-up surveillance programs [1].

The COVID-19 pandemic led to significant disruptions in colorectal cancer care within Australia, with increasing clinical demands on our healthcare system. We sought to analyse our CRC surveillance program in the context of the pandemic and to enhance our system for the future.

This study therefore aimed to assess the overall level of adherence of patients to surveillance follow-up post-surgery for non-metastatic CRC at a tertiary referral centre between January 2019 and December 2022.

## Methods

A retrospective analysis was undertaken to evaluate all patients who underwent curative resection for colorectal adenocarcinoma at Western Health between January 2019 and January 2022. Western Health, located in metropolitan Victoria, Australia, serves a population of approximately one million and is recognised as a major tertiary referral centre. Patients were excluded from the study if they did not participate in follow-up care, had their care transferred to another institution, or did not require surveillance as decided by the CRC multidisciplinary team.

Patients were identified using our prospective CRC registry (ACCORD). Patient data from the hospital Electronic Medical Records (EMR) were reviewed by two independent health practitioners to ensure data concordance. Follow-up data was recorded for the first 18-month period following CRC resection and adherence to our institutional surveillance protocol was assessed.

Patients were grouped based on their year of diagnosis as follows: 2019 (pre-COVID cohort), 2020 (during-COVID cohort) and 2021 (post-COVID cohort). These categories correspond to the dominant pandemic phases during their respective 18-month surveillance windows.

Patients’ clinical and characteristics such as age, gender and type of operation performed were recorded. Tumour characteristics, including the American Joint Committee on Cancer (AJCC) stage, were recorded for all patients.

**Fig. 1:**
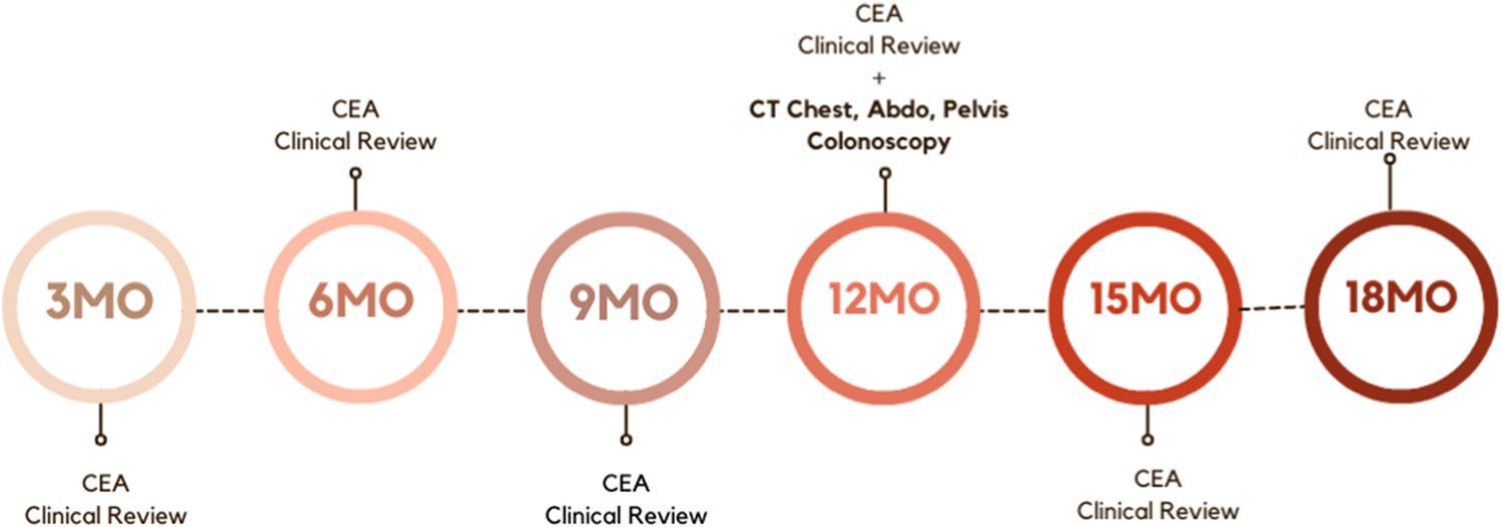
Audit standard: Surveillance protocol

Our surveillance protocol (Fig. 1) required patients to have three-monthly outpatient reviews, either in-person or by telehealth. Reviews were conducted either in a specialist-led colorectal surgical or medical oncology clinic. All patients of Non-English-Speaking Backgrounds (NESB) were provided with an interpreter during their clinic appointment.

Serum CEA was required three-monthly. Requests for CEA were completed on physical blood request forms, by providers directly linked to our hospital network. CT scans and colonoscopy were due 12 months after surgery and were requested to be completed within our own institution.

A period of 6 weeks before and after a due date for a clinical review or investigation was allowed based on findings from a previous study at our institution [25].

Descriptive data was used for patient demographics, cancer characteristics and primary procedure. Pearson chi-squared test, ANOVA, MANOVA (with post-hoc testing) and Kruskal–Wallis test (with post-hoc testing) were used as appropriate when comparing categorical variables and rates of compliance with follow-up metrics. An alpha of < 0.05 was deemed statistically significant.

Approval from the Western Health Human Research Ethics Committee was formally obtained (WH/95334/QA2023.22).

## Results

Four hundred fifty-one patients were assessed for eligibility. Seventy-one patients were subsequently excluded from follow-up (at varying stages) on the basis that they either declined follow-up (*n* = 4), were transferred to another public or private institution (*n* = 53), were deceased (*n* = 8) or were deemed to be exempt from intensive surveillance by the multidisciplinary team (*n* = 6). A total of 380 patients (Stages I, II and III) were identified, of whom 213 were male and 167 were female.

Of the 380 patients, the majority suffered from colonic cancer (70.8%). One hundred three patients presented with Stage I colorectal cancer, 129 with Stage II and 148 with Stage III. The most commonly performed primary procedure was anterior resection, *n* = 149 (39.2%). One hundred nine patients were treated in 2019, 90 in 2020, 101 in 2021 and 80 in 2022 (Table 1).

**Table 1:** Patient characteristics

Characteristics	Overall, *n* = 380 (%)	2019, *n* = 109	2020, *n* = 90	2021, *n* = 101	2022, *n* = 80
Age at time of surgery (years)	68 (31–95)	64 (35–95)	67.5 (36–91)	71 (37–87)	70 (31–89)
Median (range)					
Gender					
Male	213 (55.9)	62	48	58	45
Female	167 (44.1)	47	42	43	35
Cancer type					
Colon	269 (71.0)				
Rectal	91 (23.8)	76	61	74	
Rectosigmoid	19 (5.2)	25	24	24	18
Synchronous rectal and colon	1 (0.26)	5	5	3	61
AJCC stage					
I	103 (27.1)	30	24	22	27
II	129 (33.9)	38	36	33	22
III	148 (38.9)	41	30	46	31
Operation type					
Abdominoperineal resection	19 (5.0)				
	149 (38.9)	7	3	5	4
Anterior resection	154 (40.2)				
	R 136	49	30	35	35
Hemicolectomy				44	
Right (R)	L 18	38	38	R 41	34
Left (L)	31 (8.1)	R 32	R 35		R 28
Hartmann’s	21 (5.5)			L 3	L 6
Total or subtotal colectomy		L 6	L 3	10	
	2 (0.5)			5	
Proctocolectomy		9	9		3
TAMIS	2 (0.5)				
Pelvic exenteration		5	8	1	3
	2 (0.5)				
		1	1	1	
					1
					1

### Clinical reviews

Overall adherence with clinical reviews for Stages I, II and III CRC was 52.7%, 63.2% and 83.5%, respectively. Patients who had Stage III CRC consistently had the highest adherence at all time-points at 83.5% (*p* < 0.05) (Table 2). Stage III patients were more likely to have their surveillance with clinic reviews in comparison with Stages I and II (Chi2, *p* < 0.05).

**Table 2:** 18 months of colorectal cancer follow up 2019–2022 following curative resection. The Stage III cohort appears to be more adherent to follow-up and most likely to have recurrence picked up via surveillance. *CR* clinic review,* CEA* carcinoembryonic antigen,* mo* month, *CT* computed tomography. *% Corrected to 3 s.f

Time	(Months)	Surveillance metrics		***N* (%)**		
				Stage I	Stage II	Stage III
3mo		**CR**	***p* < 0.05**	55/98 (56.1%)	94/122 (77.0%)	113/131 (86.3%)
		**CEA**	***p* < 0.05**	*43/98 (43.9%)*	*76/123 (61.8%)*	72/129 (55.8%)
6mo		**CR**	***p* < 0.05**	57/98 (58.2%)	88/122 (72.1%)	113/129 (87.6%)
		**CEA**	***p* < 0.05**	*45/98 (45.9%)*	*64/123 (52.0%)*	89/128 (69.5%)
9mo		**CR**	***p* < 0.05**	53/98 (54.1%)	64/121 (52.9%)	105/126 (83.3%)
		**CEA**	***p* < 0.05**	*34/98 (34.7%)*	*57/122 (46.7%)*	76/125 (60.8%)
12mo		**CR**	***p* < 0.05**	51/98 (52.0%)	78/121 (64.5%)	106/126 (84.1%)
		**CEA**	***p* < 0.05**	*34/98 (34.7%)*	62/121 (51.2%)	79/124 (63.7%)
		**CT**	***p* = 0.08**	74/98 (75.5%)	92/120 (76.7%)	106/124 (85.5%)
		**Colonoscopy**	***p* = 0.57**	65/98 (66.3%)	70/117 (59.8%)	74/124 (59.7%)
15mo		**CR**	***p* < 0.05**	51/93 (54.8%)	69/117 (59.0%)	98/125 (78.4%)
		**CEA**	***p* < 0.05**	28/98 (28.6%)	54/119 (45.4%)	63/123 (51.2%)
18mo		**CR**	***p* < 0.05**	40/98 (40.8%)	64/119 (53.8%)	101/124 (81.5%)
		**CEA**	***p* < 0.05**	26/98 (26.5%)	47/119 (39.5%)	75/122 (61%)
Within surveillance period 18mo		**Recurrence and (new cancer)**	***p* < 0.05**	4 (1)/98	10 (3)/120	39 (4)/127
Post 18mo surveillance		**Recurrence and (new cancer)**		3 (0)	1 (1)	6 (0)

### Serial CEA

Overall adherence with serial three-monthly CEA levels for Stages I, II and III CRC was low at 35.7%, 49.4% and 60.3%, respectively. Patients with Stage III CRC demonstrated the highest compliance (*p* < 0.05), with a mean compliance rate of 60.3% across all time-points. There was better adherence to serial CEA levels amongst Stage III patients compared to Stages I and II (*p* < 0.05).

### CT and colonoscopy

In total, 272 patients completed their CT scan at 12 months after surgical resection (79.5%). Similar rates of compliance were seen across the three stages of CRC (*p* = 0.06). Completion of colonoscopy at 12 months was low overall, with 209 (61.7%) patients successfully undergoing the procedure within the recommended time period.

### Overall CRC outcomes

There was an overall CRC recurrence rate of 15.4% in the first 18 months of surveillance, with Stage III patients being the most likely to experience recurrence at 30.4% (*p* < 0.05), compared to 8.5% of Stage II and 6.8% of Stage I CRC.

### Impact of COVID pandemic

The COVID pandemic resulted in several lockdowns in the state of Victoria, Australia, from 2020 to 2021.

There were 116 patients in the pre-COVID group, 102 patients during COVID and 162 patients in the post-COVID. There was a higher proportion of patients who presented with Stage III CRC after COVID compared to earlier time periods: post-COVID 47.5%, compared to before COVID 35.3% and during COVID 29.4% (*p* < 0.05).

More patients underwent a 12-month CT following the COVID pandemic (87% compared to pre-COVID 73.1% or during COVID 76%) (*p* < 0.05). Adherence to CEA, clinic reviews and colonoscopy was not statistically different across the COVID timelines. Eighteen-month mortality and overall recurrence were also not statistically significant across the COVID timelines.

**Fig. 2:**
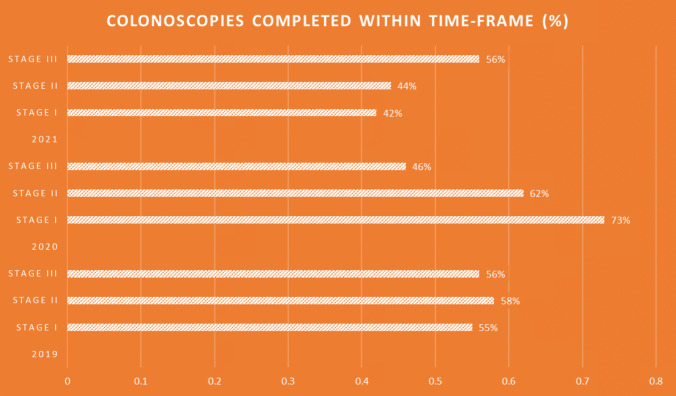
Percentage of patients who have completed their 12-month colonoscopy surveillance within the 6-week grace period

As COVID-19 restrictions affected inter-personal clinical interactions, an appropriate surrogate that combines both clinical assessment and invasive procedural investigation would be the 12-month colonoscopy surveillance. Figure 2 illustrates the percentage of patients who completed colonoscopy surveillance within the recommended time frame, stratified by year of diagnosis (2019–2021) and cancer stage (I–III). In 2019, completion rates were relatively consistent across stages (Stage I, 55%; Stage II, 58%; Stage III, 56%). In 2020, Stage I patients showed a notably higher adherence (73%) compared to Stages II (62%) and III (46%).

## Discussion

Colorectal cancer surveillance is an essential aspect of survivorship care. Current Australian national guidelines recommend that those who undergo resection for colorectal cancer with curative intent, and who are fit for further intervention, should receive intensive follow-up [3]. Some systematic reviews and meta-analyses have noted an overall survival benefit with intensive surveillance protocols [10, 11]. However, a recent, updated systematic review by Jeffery et al., which included over 13,000 patients, did not find any improvement in overall survival, disease-free survival, or relapse-free survival with intensive CRC surveillance regimens [12]. This finding is in keeping with the analysis of data from the National Cancer Database [13]. Nevertheless, current Australian guidelines recommend intensive surveillance for suitable patients who undergo resection for colorectal cancer with curative intent [3].

Surveillance typically involves a combination of clinical evaluation and investigations including serial serum carcinoembryonic antigen (CEA) measurement, computed tomography (CT) scans and colonoscopy. Such regimens are often time and resource-intensive for healthcare systems. Due to increasing demand on health networks and variation in patient background demographics and understanding of the importance of surveillance follow-up, adherence to these guidelines can be highly variable [4].

This study aimed to identify if survivorship care at our institution changed due to the pandemic and how a subsequent increase in clinical demand might have impacted our surveillance provision.

Approximately 50% of our patients completed serial serum CEA tests. Additionally, colonoscopy provision at 12 months was lower than previously published Australian data [14, 15]. CT scans performed at the 12-month period had the greatest adherence of all the investigations.

We found that Stage III patients had the highest rates of compliance to the surveillance protocol for all tests; clinical review reached 90% uptake. This may be explained by the fact that higher stage patients who also had medical oncology treatment were more likely to have multiple survivorship review requests by the multidisciplinary team or had a better understanding of the importance of surveillance take-up.

There was a change in how clinical follow-up was offered during the pandemic, similar to many other health institutions within Australia. Patients during the pandemic were offered “telehealth” (mainly telephone) appointments and this has continued to the present day. There is evidence in the literature that the disruptions caused by the pandemic have led to reductions in screening, diagnostic and treatment services for colorectal cancer both nationally and internationally [22]. From our study, we have shown that adherence to our CRC surveillance was largely unaffected by the COVID pandemic.

Our study also showed that a greater proportion of our patients presented as Stage III cancers after COVID. This is no different to other national and international data where in particular screening colonoscopies and patient presentation of symptoms were impacted by the pandemic [23, 24, 26].

There are several learning points from this study. There are still barriers with regard to institutional workflows and patient characteristics which have led to the variation in survivorship care between different cancer stages. We have shown that certain aspects of surveillance protocol adherence have the potential for high uptake such as in-person clinical reviews. This may necessitate in-person clinician-patient interaction before other investigations or as a follow-up of results; we hope this would then optimise adherence to investigations.

Utilising nursing staff roles to incorporate tasks previously undertaken by medical staff has been suggested to expedite healthcare delivery in cancer care, as well as to aid in healthcare cost reduction [17, 18]. Nurse-led clinics for patients with other malignancies, such as breast, lung and prostate, have been reported to be both successful and well received by patients [19, 20]. In the Australian context, Moloney et al. were able to achieve overall compliance of 97.4% and high patient satisfaction with a nurse-led CRC surveillance program over a 10-year period [21]. Within our institution, we designed a nurse–led cancer surveillance program, utilising patient tracking software which aims to reduce duplication of requests, streamline workflows and ensure timelier survivorship follow-up. This initiative was implemented after the conclusion of our study period (2019–2022). As such, its impact on adherence is not reflected in the current dataset; however, future studies will explore its role in improving compliance.

Our study has several limitations. It is a retrospective, and as such, the results were dependent on the quality of the original raw data. However, all patients treated surgically for CRC had been included in our prospectively maintained registry. Although the sample size was relatively small, and patients were only followed up for an 18-month period, we have identified information that we can use to address certain areas in our future practice. Finally, while we examined uptake of surveillance, we did not examine factors that may have contributed to these results.

We also acknowledge that Western Melbourne has an incredibly diverse population, and more than 30% of the community is born outside of Australia. Within our catchment, at least 110 different languages are spoken, with around 8% of the total population having poor or no English proficiency, significantly higher than the national average of 2.6% [27]. We know patients from Culturally and Linguistically Diverse (CALD) backgrounds have unique barriers to healthcare and, as such, deserve a dedicated review [28, 29].

## Conclusion

Survivorship care is an essential aspect of colorectal cancer treatment. Within our institution, patients with different cancer stages had different surveillance program adherence. Further work is required to identify how we can improve our survivorship care for these patients.

## Data Availability

No datasets were generated or analysed during the current study.
